# Anti-aging mechanism of different age donor-matched adipose-derived stem cells

**DOI:** 10.1186/s13287-023-03415-3

**Published:** 2023-08-02

**Authors:** Tao Wang, Yingyu Li, Yu Zhu, Zebiao Liu, Li Huang, Hongxia Zhao, Zuping Zhou, Qiong Wu

**Affiliations:** 1 Guangxi Universities Key Laboratory of Stem Cell and Biopharmaceutical Technology, Research Center for Biomedical Sciences, School of Life Sciences, Guangxi Normal University, Guilin, 541004 China; 2 Faculty of Biological and Environmental Sciences, University of Helsinki, 00014 Helsinki, Finland

**Keywords:** Adipose stem cells (ASCs), Aging, Obesity, Multi-omics

## Abstract

**Background:**

Adipose-derived stem cells (ASCs) have anti-aging and anti-obesity effects in aged animals, but the underlying molecular mechanism remains unknown.

**Methods:**

In the present study, we evaluated the in vivo transplantation effects of different age donor-matched ASCs on natural aging and leptin knockout mice (ob^−^/ob^−^ mice). The multi-omics expression profiles of young and aged mouse donor-derived ASCs were also analyzed.

**Results:**

The results revealed that ASCs from young donors induced weight and abdominal fat loss for older recipients but not for young or ob^−^/ob^−^mice. The young and aged mouse donor ASCs displayed significant phenotypic differences, contributing to the distinguished weight loss and anti-aging effects in aged mice.

**Conclusions:**

Our data suggest an underlying molecular mechanism by which young-donor ASCs reduce immune cells and inflammation in aged mice via secreted immune factors. These findings point to a general anti-aging mechanism of stem cells, which may provide new insights into age-related disturbances of stem cell plasticity in healthy aging and age-related diseases.

**Supplementary Information:**

The online version contains supplementary material available at 10.1186/s13287-023-03415-3.

## Introduction

Adipose tissue is a highly active metabolic and endocrine organ whose functions decline with aging and obesity [13]. Changes in the distribution and function of adipose tissue occur throughout an individual's life. Adipose tissue undergoes dramatic changes in various aspects during aging, mainly manifested as reduced subcutaneous fat but elevated visceral fat in the abdomen [21, 24]. Increased abdominal adipose tissue is the primary phenotypic characteristic of obesity with aging and is also associated with many adverse consequences, such as hypertension, type 2 diabetes (T2D), cardiovascular disease, etc. [22].

Adipose-derived stem cells (ASCs) are functional cells in adipose tissue that belong to mesenchymal stem cells, accounting for about 10–20% of the cells in adipose tissue. ASCs have highly expressed CD13 + , CD29 + , CD44 + , CD73 + , CD90 + , CD63 + , CD105 + , and other surface markers that can participate in maintaining the dynamic balance of adipose tissue through self-renewal and differentiation into multiple cell lineages [3]. As a cell-based therapy, ASCs receive increasing attention in tissue repair, immune and endocrine regulation, anti-aging and –obesity [1]. For example, the autologous adipose-derived stem cells can regulate blood content, renal function, numbers of biochemical parameters, and expression of antioxidant enzymes in aged rats [20] ASC treatment also improved aging-related erectile dysfunction [25]. However, the performance of ASCs is not constant, with changes in different physiological states of donors. Many studies have demonstrated that the ASCs’ characteristics are affected by aging. A donor-matched comparison indicated that the properties of ASCs have significant variation between donors, which may influence the stem cell-based therapy results [15]. Especially for autologous adipose stem cell transplantation, the potential differentiation and tissue repair ability of aging ASCs are weakened, which limits the transplantation effect [10].

In agreement with previous findings in humans and other animals [2, 10], we observed an age-dependent impairment of ASCs including increased senescent and apoptosis, reduced osteogenic differentiation between young (2 months) and old (22 months) C57BL/6 J mice [28]. However, the molecular mechanisms underlying the anti-aging effects of different age donor-matched ASCs are complex and are not yet fully understood.

In the present study, we evaluated the transplantation effects and multi-omics expression profiles of young and aged mouse-donor derived ASCs to reveal the molecular mechanism by which ASCs associate with aging.

## Materials and methods

### Animal preparation and transplantation

C57BL/6 J mice were obtained from the experimental animal department of stem cells, Guangxi Normal University (Guilin, China), and leptin knockout C57BL/6 J mice (ob^−^/ob^−^ mice) were purchased from GemPharmatech Co., Ltd (Nanjing, China). CRISPR/cas9 technology and blastocyst injection were used to delete the coding regions of exon2 and exon3 of the mouse LEP gene to generate a mouse model with a frameshift mutation in the LEP gene. The study was performed in accordance with the protocols approved by the guidelines for the care and use of animals at Guangxi Normal University, and the present experiment was approved by the animal ethics committee at Guangxi Normal University ((1) Title of the approved project: Study on the inhibitory effect of adipose stem cell transplantation on age-related obesity; (2) Approval number: 20201009–001; (3) Date of approval October 9, 2020). All animals were housed under specific pathogen free conditions with 12-h light/dark cycles at 23 °C (± 2 °C) and use of standard chow diet (10% lipids) with free access to water and food.

The young and aged mouse models were established similarly to our previous method, with young mice being 2 months old and aged mice being 22 months old. 30 aged mice were randomly divided into three groups of 10 each, and the mice in the experimental group were injected with 10^6^ Y-ASCs or O-ASCs intraperitoneally, and the mice in the control group were injected with phosphate buffer (PBS, SANGON biotech, China); Similarly, 30 young mice were randomly divided into three groups of 10 each, and the mice in the experimental group were injected with 10^6^ Y-ASCs or O-ASCs intraperitoneally, and the mice in the control group were injected with PBS. Twelve ob^−^/ob^−^ mice were randomly divided into three groups (four mice per group) and treated with 10^6^ Y-ASCs and O-ASCs via intraperitoneal injection, while the control group was injected with PBS.

### ASCs and lymphocyte cultures

ASCs were isolated and cultured similarly to previous studies. Briefly, Mice were euthanized through intraperitoneal injection of urethan (1350 mg/kg) through intraperitoneal injection of urethane (1350 mg/kg). Adipose was obtained from the groin of mice using sterile scissors and tweezers, and the cleaned fat was cut into small pieces with a side length of about 1 mm on the super clean workbench (ESCO, Singapore). After fat pieces were digested with an equal volume of 0.1% collagenase type I (GIBCO, America) at 37 °C for 30 min, the digestion was terminated and washed twice using DMEM/F12 (GIBCO, China) medium containing 10% fetal bovine serum (GIBCO, Australia), followed by the use of 70 μm cell strainer, centrifuged at 300 × *g* and resuspended using the above medium to obtain the vascular matrix components, which were placed into six well cell culture plates (Corning, China) with ASCs adhering to the bottom and passaged when cells were 80% full of the bottom. Cells at the third passage were used for experiments.

The mouse spleen was taken and placed in pre-cooled PBS at 4 °C. Crush it with scissors. The spleen tissue suspension was taken in a 15 ml centrifuge tube. 300 g, 5 min, remove the supernatant, re-suspended with five times the volume of red blood cell lysate. After lysis at 4 °C for 30 min, the lymphocyte separation solution was added, 300 g, 8 min, and the intermediate lymphocyte layer was cultured in 1640 (GIBCO, China) supplemented with 20 ng/ml Concanavalin A (Solarbio, China).

### Detection of body weight, tissue weight and aging markers

The weight of mice was weighed weekly during the month after ASCs transplantation, and the daily food intake of mice was recorded. One month later, the mice were dissected, and the total abdominal fat weight of the mice was weighed. Mouse whole blood was collected by eye enucleation for 2 h at room temperature and then centrifuged at 1000*g* for 20 min (Hitech, ct15re) according to the supplier's instructions (mlbio, China), and the serum levels of superoxide dismutase (SOD), catalase (CAT), thyroid stimulating hormone (TSH), and sex hormone binding globulin (SHBG) were measured using ELISA kits (mlbio, China).

### Transcriptomics and proteomics

ASCs in good growth conditions were washed once using sterile enzyme-free PBS, and then appropriate amounts of triquick reagent (Solarbio, China) were added by pipetting repeatedly several times to complete lysis. Detection of gene expression was performed by the Collaborator using the method of next-generation sequencing (NGS) (Illumina sequencing platform). We performed transcriptomic sequencing of adipose stem cells from young and aged mice, as well as abdominal adipose from control and experimental groups of mice transplanted with adipose stem cells in a total of three replicates per sample.

According to the protocol of the Collaborator (Metvare, China), we provided adipose stem cells derived from young and aged mice, and the coauthor used tandem mass tags (TMT) technology to label proteins, which were quantified by mass spectrometry.

### Construction and analysis of PPI networks and functional annotation

We used the online tool STRING (http://www.string-db.org) to construct interactome maps of changed genes. We used STRING to map the identified common DEGs into the PPI network, which was then visualized by Cytoscape. The indicated network properties include: Nodes: number of proteins in the network; Edges: number of interactions; Node degree: average number of interactions; Clustering coefficient: indicates the tendency of the network to form clusters. The closer the local clustering coefficient is to 1, the more likely it is for the network to form clusters; PPI enrichment p value: indicates the statistical significance. Proteins are considered hubs when they have more interactions than the average (nº interactions > node degree). The Molecular Complex Detection (MCODE) plug-in was utilized to select the significant blocks from the PPI network, with cut-off criteria of degree ≥ 2, node score ≥ 0.2, K-core ≥ 2, and max depth = 100.

### RT-PCR

Total RNA from cells or tissues was extracted using triquick reagent (Solarbio, China). In brief, cells were lysed using triquick reagent or tissues were grounded to obtain tissue homogenate, followed by centrifugation, isopropanol was added to obtain a pellet, and total RNA was dissolved in 75% ethanol after several washes with DEPC treated water. The resulting total RNA was reverse transcribed according to the supplier's (Beyotime, China) protocol, and RT-PCR was performed according to the supplier's procedure (Absin, China). The primer sequences are shown in Additional file 5: Table S4.

### Immunoassay

We examined the abundance of adipose T cells, B cells, and NK cells in the abdominal fat of aging mice after ASCs transplantation using flow cytometry, and detailed antibody labeling is shown in Additional file 2: table s1. Interleukin-6 (IL-6), tumor necrosis factor α (TNF-α) in aged mice after transplantation of Y-ASCs, and Interleukin 1β (IL-1β) were detected using an ELISA Kit (mlbio, China).

### ASCs/lymphocyte co-culture

3 × 10^4^ Y-ASCs cells were seeded on 96-well plates. After 24 h, 3 × 10^5^ lymphocytes were inoculated on the upper layer of Y-ASCs and co-cultured for 48 h.

### Data processing

Results are presented as mean (± SD) with the exception of transcriptomics and proteomics. P was calculated using *t*-test, One-Way ANOVA or Two-Way ANOVA analysis of variance and considered significant at *P* < 0.05. Figures were plotted using graphpad prism 9.0 software (graphpad).

Using *P* < 0.01 as a criterion, we filtered out genes with differential expression. In transcriptome, we performed an analysis of 50 genes that were elevated and 50 genes that were decreased compared to the controls. In the combined cytomics analysis, we analyzed all the common differentially-expressed genes. The heat maps were made with graphpad prism 9.0, and normalization was performed with the Z-score method. Enrichment analysis and visualization of go and KEGG were performed using R 3.6.3 software (clusterprofiler package for enrichment analysis and the ggplot2 package for visualization).

## Results

### The effects of transplantation of age-donor matched ASCs for different recipients

**Fig. 1:**
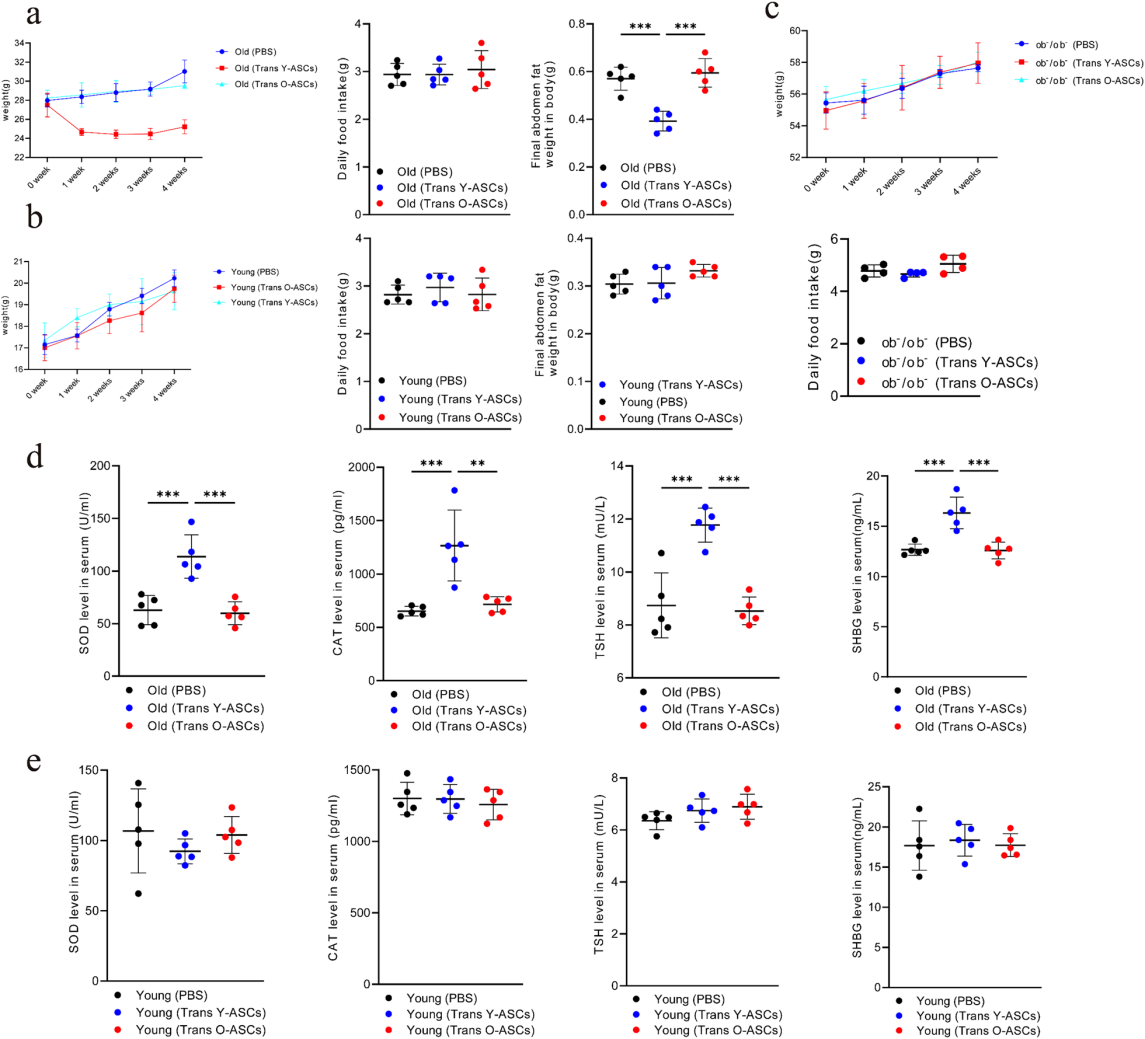
Effects of Y-ASCs and O-ASCs transplantation 1 month on young mice, aged, and ob^−^/ob^−^ mice. **a** Effects of Y-ASCs and O-ASCs transplantation on body weight, food intake, and abdominal fat proportion in aged mice. **b** Effects of Y-ASCs and O-ASCs transplantation on body weight, food intake and abdominal fat proportion in young mice. **c** Effects of Y-ASCs and O-ASCs transplantation on body weight and food intake in ob^−^/ob^−^ mice. **d** The expression levels of superoxide dismutase (SOD), catalase (CAT), thyroid stimulating hormone (TSH), and sex hormone-binding globulin (SHBG) in the serum of aged mice transplanted with Y-ASCs and O-ASCs. **e** The expression levels of SOD, CAT, TSH and SHBG in the serum of young mice transplanted with Y-ASCs and O-ASCs. All data are mean ± SD with n = 5 for old mice, n = 5 for young mice and n = 4 for ob^−^/ob^−^ mice. **P < 0.01, ***P < 0.001

Our previous study showed that transplantation of young mouse ASCs (Y-ASCs) reduced body weight and abdominal adipose tissue in aged mice [31]. However, the effects of ASC transplantation from old donors (O-ASCs) are yet to be defined. Thus, we compared the effects of Y-ASCs and O-ASCs transplantation on body weight, food intake, and abdominal fat proportion in aged mice (Fig. 1a). In line with our previous findings, aged mice treated with Y-ASCs had significantly lower body weight and abdominal fat after one month, whereas aged mice treated with O-ASCs had the same weight and abdominal fat content as the control mice.

In order to explore the effect of Y-ASCs transplantation is related to age or degree of obesity, leptin-deficient ob/ob mice were choosed for morbidly obese. We examined both Y-ASCs and O-ASCs transplantation in young and leptin knockout mice (ob^−^/ob^−^ mice). The results showed that the effects of transplantation on body weight, food intake, and abdominal fat proportion of young and ob^−^/ob^−^mice were similar in all groups (Fig. 1b and c). However, the ASCs from young donors have a significant impact on weight and abdominal fat loss for older recipients (Fig. 1a). It is generally accepted that the production of antioxidant enzymes and hormones declines with age. Thus, to examine whether the effects of Y-ASCs transplantation are related to anti-aging, we compared the changes of age-related antioxidant enzymes and hormones after transplantation of Y-ASCs and O-ASCs. The results revealed that the levels of SOD (Superoxide Dismutase), CAT (Catalase), TSH (Thyroid Stimulating Hormone), and SHBG (Sex Hormone Binding Globulin) in the serum of aged mice treated with Y-ASCs were significantly increased by 81.02%, 79.31%, 34.69%, and 32.15%, respectively (Fig. 1d). However, O-ASCs had no effect on their abundance in the aged mice (Fig. 1d). The levels of SOD, CAT, TSH, and SHBG were similar in all groups of young mice (Fig. 1e). These results indicate that only Y-ASCs have anti-obesity and anti-aging effects on aged mice.

### Gene expression profiles of young and aged mouse donor-derived ASCs

The ASCs from young and old mouse donors have significant variations, which may influence the stem cell-based therapy results. To explore the underlying molecular mechanism, we analyzed the transcriptome and proteome of Y-ASCs and O-ASCs.

**Fig. 2:**
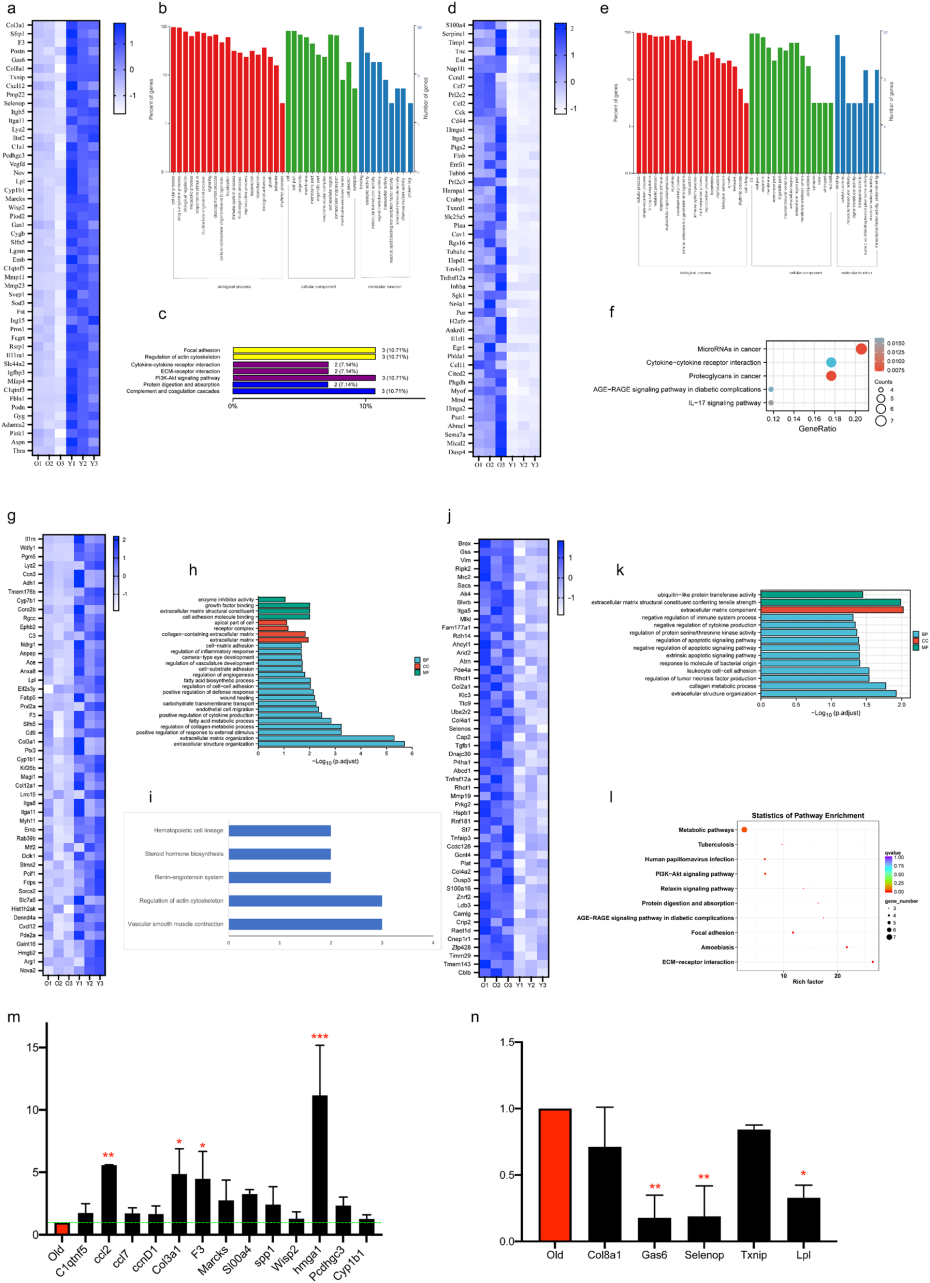
Analysis and verification of Y-ASCs and O-ASCs transcriptome and proteome. **a** The heatmap of Top50 genes highly expressed in the Y-ASCs. **b**. GO functional classification analysis of top50 genes highly expressed in the Y-ASCs. **c** KEGG classification analysis of top50 genes highly expressed in the Y-ASCs. **d** The heatmap map of Top50 genes highly expressed in the O-ASCs. **e** GO functional classification analysis of top50 genes highly expressed in the O-ASCs. **f** KEGG bubble diagram of the O-ASCs with highly expressed top50 genes. **g** The heatmap of top50 proteins highly expressed in the Y-ASCs compared with O-ASCs. **h** GO functional classification analysis of top50 proteins highly expressed in the Y-ASCs. **i** KEGG classification analysis of top50 proteins highly expressed in the Y-ASCs. **j** Compared with the Y-ASCs, top50 protein thermograms highly expressed in the O-ASCs. **k** GO functional classification analysis of top50 proteins highly expressed in the O-ASCs. **l** KEGG bubble diagram of top50 proteins highly expressed in the O-ASCs. **m** Expression of inflammation related genes verified by RT-PCR. **n** Verification of fatty acid related gene expression by RT-PCR. Each bar represents the difference change of genes expression in Y-ASCs with O-ASCs as calibrator. Expression levels were normalized to that of gene GAPDH. O: O-ASCs; Y: Y-ASCs; All data are mean ± SD, n = 3 ~ 5 for each group

Our data showed that a total of 1667 genes were differentially expressed in the Y-and O-ASCs (false discovery rate < 0.05 and folder-change ≥ 2, Additional file 1: Fig. S1, Additional file 2: Table S1). Compared to the O-ASCs, 686 up-regulated and 981 down-regulated mRNAs were found in the Y-ASCs (Additional file 1: Fig.S1). The results indicated that Y-ASCs and O-ASCs were distinct in gene expression. These differentially expressed genes were significantly enriched in fatty acid metabolism and inflammatory response (Fig. 2b and e), and pathways in cytokine-cytokine receptor interaction, complement, and others (Fig. 2c, f). To validate the transcriptome data, some genes associated with the fatty acid metabolism and inflammatory response were chosen for analysis by real-time RT-PCR, including C1q, Ccl2, Ccl7, CcnD1, F3, Hmga1, Gas6, Selenol, Txnip, Lpl, and others. Among these genes, higher expression of Ccl2, F3, and Hmga1 (*P* < 0.05) but lower expression of Gas6, Selenol, and Lpl (*P* < 0.05) were observed in the Y-ASCs as compared to the O-ASCs, confirming the transcriptome results (Fig. 2m and n).

Furthermore, the proteome data indicated that a total of 268 proteins were differentially expressed in the Y-ASCs and O-ASCs (false discovery rate < 0.05 and folder-change ≥ 2, Additional file 1: Fig. S2; Additional file 3: Table S2). There were 155 up-regulated and 113 down-regulated proteins in the Y-ASCs compared to the O-ASCs (Additional file 1: Fig.S2). The heatmap of the highest expression difference and the most abundant top50 proteins in the Y-ASCs are listed in Fig. 2g. In line with the transcriptome analysis, the GO and KEGG analysis further revealed that these upregulated proteins were involved in fatty acid metabolism and inflammatory response (Fig. 2h and i). The heatmap of the lowest expression difference and the most abundant top50 proteins in the Y-ASCs are listed in Fig. 2j. The GO and KEGG analysis revealed that these down-regulated proteins were significantly enriched in apoptotic signaling and metabolic pathways (Fig. 2k and l). This is consistent with our previous findings in the characteristics of Y-ASCs and O-ASCs [28], in which O-ASCs increased senescence and apoptosis while decreasing osteogenic differentiation.

**Fig. 3:**
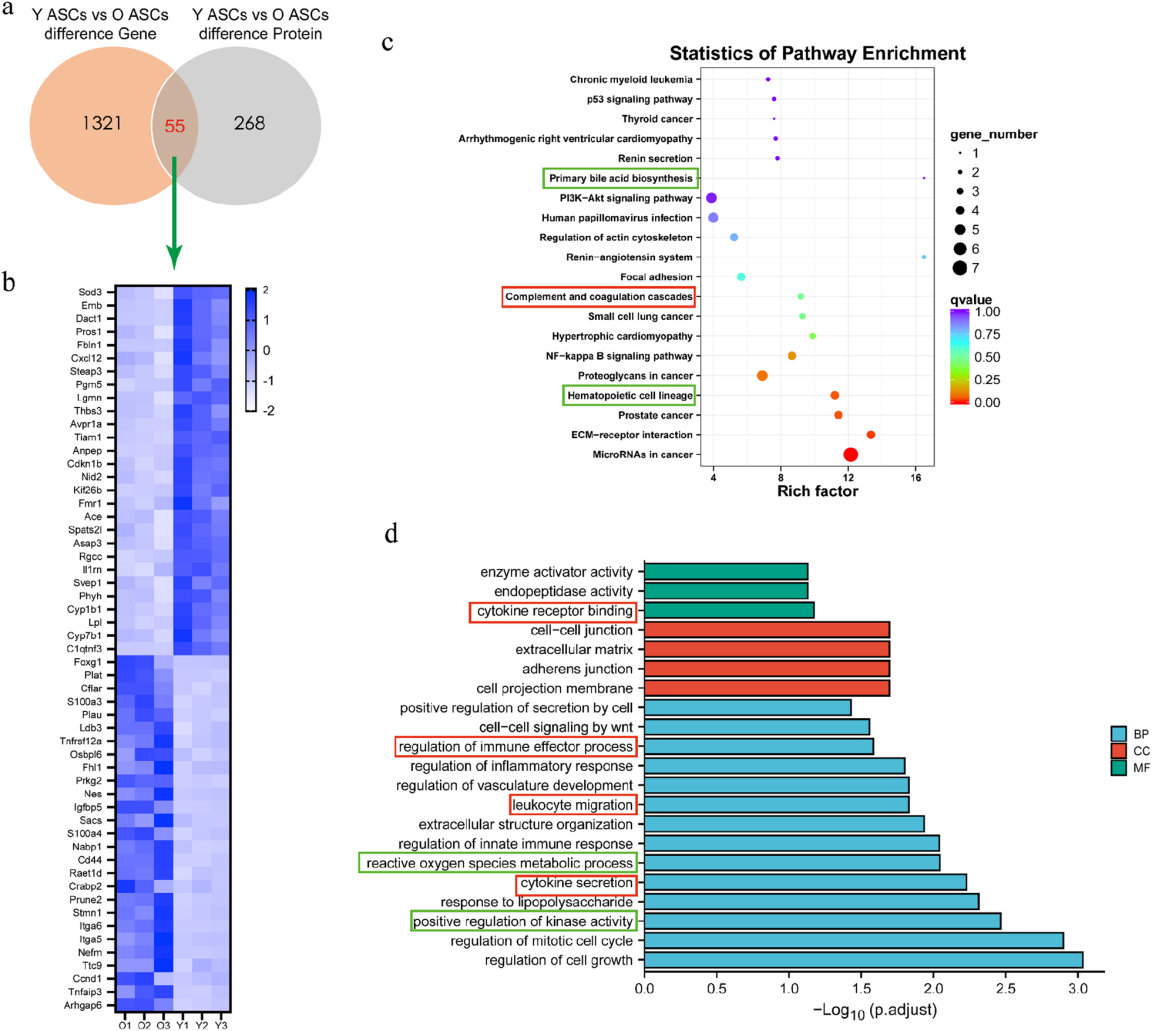
Combined analysis of transcriptome and proteome of Y-ASCs and O-ASCs. **a** Venn diagram showing there are 55 overlaps between the transcriptome and proteome. **b** Heatmaps of the 55 overlapped proteins. **c** KEGG bubble diagrams of the 55 overlapped proteins. **d** GO classification analysis of the 55 overlapped proteins. O: O-ASCs; Y: Y-ASCs; n = 3 for each group

Furthermore, the Venn analysis revealed 55 genes that differed in common between the transcriptome and proteome (Fig. 3a), including 28 up-regulated and 27 down-regulated proteins in the Y-ASCs compared to the O-ASCs (Fig. 3c). The GO and KEGG analysis showed that these genes were significantly enriched in cytokine secretion, fatty acid metabolism, and inflammatory response (Fig. 3b and d). The omics data suggest that the expression profiles of the ASCs have significant variation between the young and old mouse donors. The differential genes between the Y-ASCs and O-ASCs are related to immunity and adipogenicity, which may influence the stem cell-based therapy results.

In light of our recently labeled ASCs for tracing, only a handful of cells survived after seven days of transplantation [31]. Therefore, the effects of cell transplantation are mainly caused by secreted proteins or small molecules induced by stem cells, consistent with other reports [4, 17, 18]. Moreover, the in vivo transplantation showed that only Y-ASCs transplantation reduced the body weight and abdominal adipose tissue in aged mice.

### The expression profiles of adipose tissue in aged mice transplanted with ASCs

**Fig. 4:**
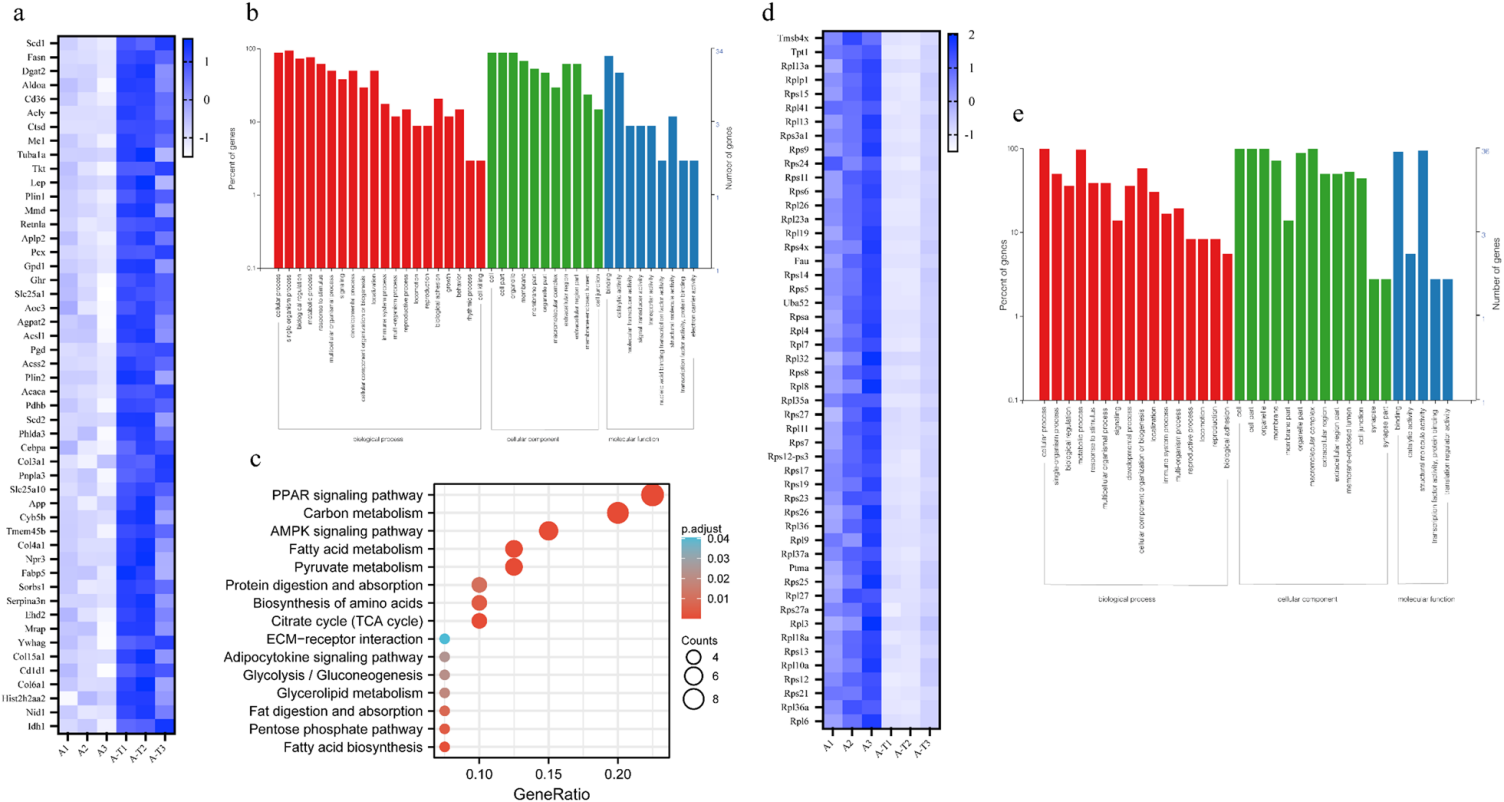
Transcriptome analysis of aged mice receiving the Y-ASCs transplantation. **a** The heatmap of top50 up-regulated genes in adipose tissue of aged mice transplanted with Y-ASCs. **b** GO functional classification analysis of up-regulated top50 gene expression in the adipose tissue of aged mice transplanted with Y-ASCs. **c** KEGG bubble diagram of up-regulated top50 gene expression in the adipose tissue of Y-ASCs transplanted aged mice. **d** The heatmap of down-regulated top50 genes in the adipose tissue of aged mice transplanted with Y-ASCs. **e** GO functional classification analysis of down-regulated top50 gene expression in the adipose tissue of Y-ASCs transplanted aged mice. A: adipose tissue of aged mice; A-T: adipose tissue of aged mice transplanted with Y-ASCs; n = 3 for each group

The previous transplantation experiment showed that only Y-ASCs reduced the weight and abdominal fat of aged mice. To clarify the molecular changes in abdominal fat in aged mice after the Y-ASCS transplantation, transcriptome sequencing of the abdominal adipose tissue was performed. The results showed that a total of 2161 genes were differentially expressed in the abdominal adipose tissue of Y-ASC transplanted old mice compared to the control, including 1024 up-regulated and 1137 down-regulated mRNAs (false discovery rate < 0.05 and folder-change ≥ 2, Additional file 1: Fig.S3, Additional file 4:Table S3). The heatmap of the most differentially expressed and abundant top50 genes in the Y-ASCs-transplanted old mice are shown in Fig. 4a. The GO and KEGG analysis revealed that these genes are significantly enriched in various fat-forming and metabolism pathways, and immune system responses (Fig. 4b and c). These transcriptome results indicate that Y-ASCs transplantation alters the abdominal fat metabolism as well as immune state in aged mice.

### Identification of Hub genes in protein–protein interaction network

**Fig. 5:**
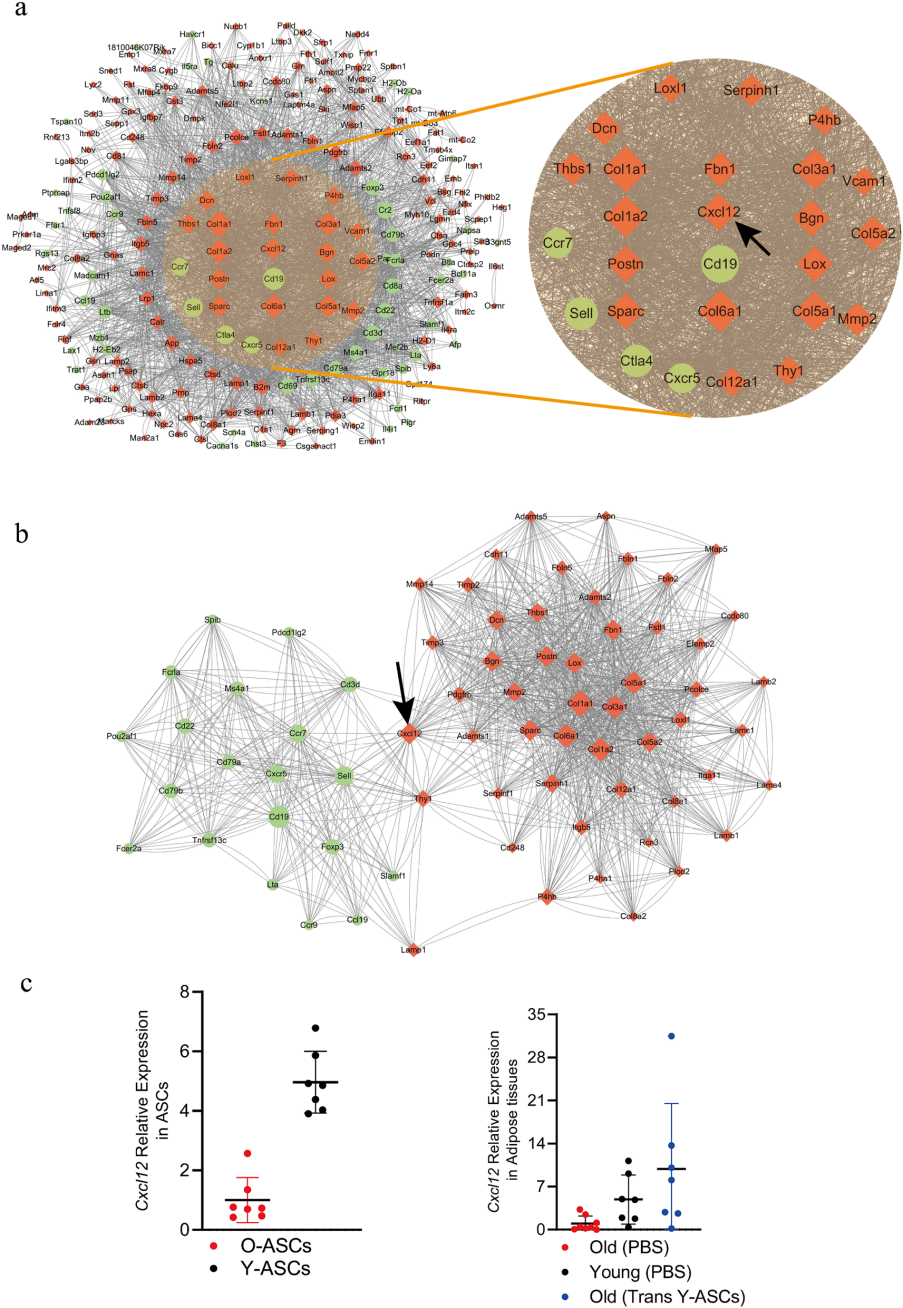
Protein–protein interaction (PPI) network of differentially expressed genes. **a** Identification of hub genes. Top 200 genes whose expression was up-regulated in the Y-ASCs were analyzed using O-ASCs as control (marked as red). 79 genes were identified as commonly down-regulated in the young and aged adipose tissues after transplantation of Y-ASCs, using aged adipose tissues as control (marked as green). **b** The important nodes in the PPI network found by MCODE plug-in. **c** The transcriptional levels of Cxcl12 verified in different aged ASCs and adipose tissues. All data are mean ± SD, n = 7 ~ 8 for each group

To build a protein–protein interaction (PPI) network and identify the potential functional modules in the network, the top 200 genes whose expression was up-regulated or down-regulated after transplantation of Y-ASCs were chosen for analysis using the online tool STRING. Notably, compared to the PPI network of upregulated genes in the young (average node degree: 3.49; clustering coefficient: 0.389; Additional file 1: Fig.S4 a) and aged adipose tissues (average node degree: 2.9; clustering coefficient: 0.441; Additional file 1: Fig. S4b), the PPI network of downregulated genes in the young (average node degree: 10.6; clustering coefficient: 0.467; Additional file 1: Fig. S4c) and aged adipose tissues after transplantation of Y-ASCs (average node degree: 5.21; clustering coefficient: 0.4; Additional file 1: Fig. S4d) displayed more concentrated clusters. Similar biological processes and molecular function enrichment in the PPI network of downregulated genes in the young and aged adipose tissues after transplantation of Y-ASCs were found, such as CD8-positive, gamma-delta intraepithelial T cell differentiation, immune receptor activity, and cytokine receptor activity. Among them, 79 genes were commonly down-regulated in the young and aged adipose tissues after transplantation of Y-ASCs (Additional file 1: Fig. S4e). In order to understand which types of genes are highly expressed in the Y-ASCs, which lead to similar genomic changes in the young and post-transplantation mice, we analyzed the top up-regulated 200 genes in the Y-ASCs, using O-ASCs as a control. The 79 genes down-regulated in the young and aged adipose tissues after transplantation of Y-ASCs were combined for PPI network analysis (average node degree: 10.7; clustering coefficient: 0.468), which identified 275 nodes (proteins) and 1474 edges (interactions). 40 of the 279 genes were filtered out and nodes that have the most interactions were considered as hub genes. Among the 239 nodes, 26 were identified as the hub genes with the criteria of node degree ≥ 60 (Fig. 5a). Notably, many of these hub genes, such as Col1a1, Col1a2 [9], Col3a1 [12], Col5a1 [29], and Bgn [5] are associated with inflammatory responses. Furthermore, the molecular complex detection (MCODE) plug-in was applied to select the significant blocks in the PPI network. The important areas with the highest scores were screened out consisting of 71 nodes and 1434 edges (Fig. 5b). It is worth noting that Cxcl12 located in the central position and its expression was validated by RT-PCR (Fig. 5c).

### Changes of the immune environment of adipose tissue in aged mice after Y-ASCs transplantation

**Fig. 6:**
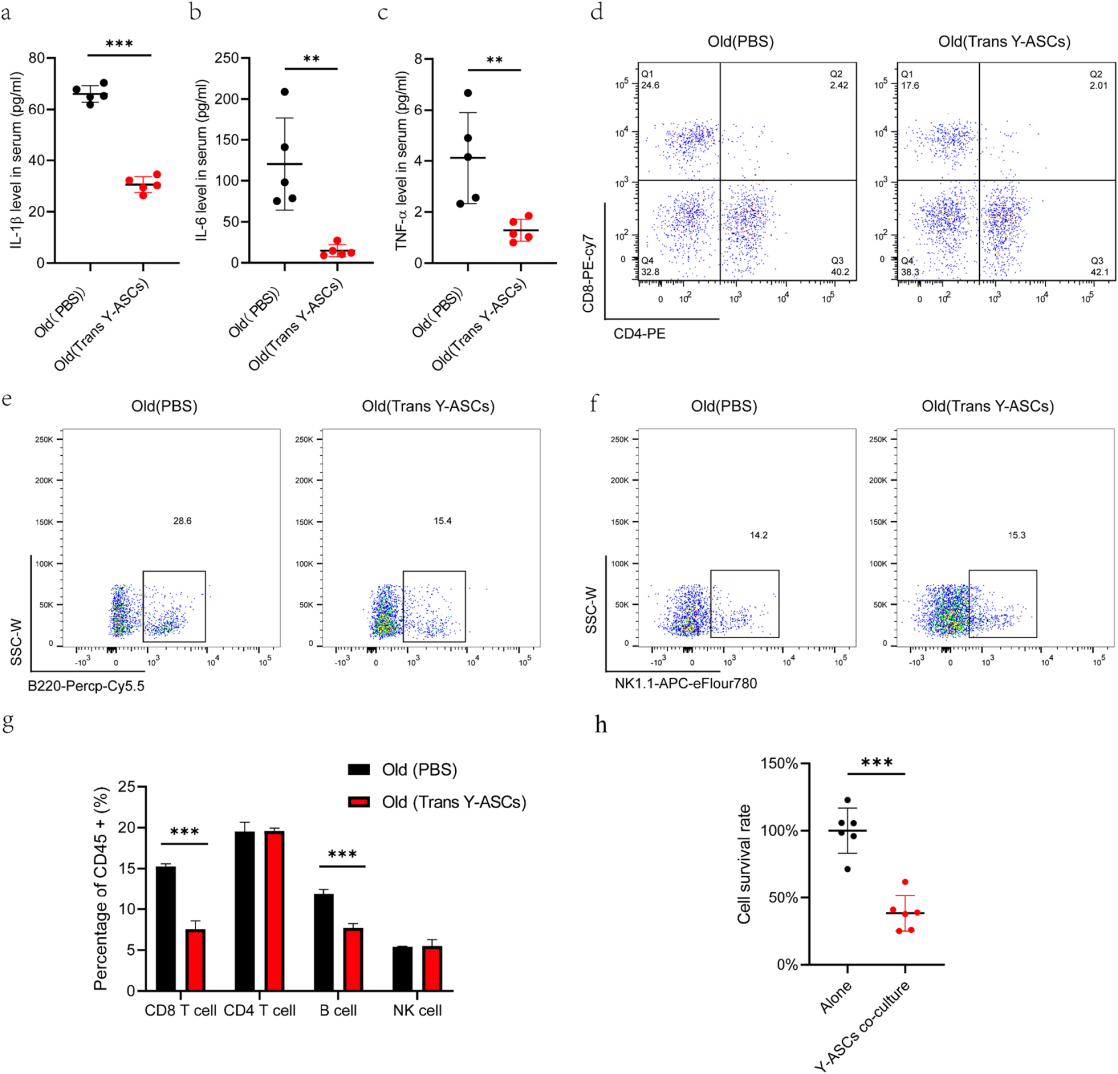
Transplantation of Y-ASCs alleviates chronic inflammation caused by natural aging. **a,b,c** represent the levels of IL-1β, IL-6, and TNF-α in serum were significantly decreased in the Y-ASC transplantation group when compared to the old control group. **d** The T cells in adipose tissue of aged mice after the transplantation of Y-ASCs when compared to the old control group by flow cytometry. **e** The B cells in adipose tissue of aged mice after the transplantation of Y-ASCs by flow cytometry. **f** The NK cells in adipose tissue of aged mice after the transplantation of Y-ASCs by flow cytometry. **g** Quantification of lymphocyte changes in adipose tissue of aged mice after the transplantation of Y-ASCs. The killer T cells (CD3 + CD8 +) and B cells were significantly decreased in the Y-ASC transplantation group. The helper T cells (CD3 + CD4 +) and natural killer cells (NK cells) were not changed. **h** The proliferation of lymphocytes with Y-ASC co-cultured was detected by CCK-8. All data are mean ± SD with n = 5. **P < 0.01, ***P < 0.001

According to a recent report on the role of immune cells and their secreted cytokines in obesity, adipogenesis, and metabolic disorders [7], we analyzed the immune cell distribution and inflammatory cytokines in aged mice after Y-ASCs transplantation. The results showed that the levels of IL-1β, IL-6, and TNF-α in serum were reduced by 68.72%, 87.69%, and 53.70%, respectively (Fig. 6a–c). Cell sorting of the abdominal adipose tissue by flow cytometry showed that Y-ASCs transplantation significantly reduced the killer T cells (CD3 + CD8 + cells) by 7.72% and the B cells by 4.18%. The numbers of helper T cells (CD3 + CD4 + cells) and natural killer cells (NK cells) were not changed significantly (Fig. 6d–g). Therefore, we isolated lymphocytes from mouse spleen and co-cultured with Y-ASCs. The results showed that Y-ASCs significantly inhibited lymphocyte proliferation. These results suggest that Y-ASCs transplantation can alleviate chronic inflammation caused by aging and reduce the immune response of abdominal adipose tissue.

## Discussion

ASCs are important regulators of inflammation/immune response and have great therapeutic potential in cell-based treatment of aging and obesity diseases. Human adipose-derived stem cells (hASCs) from obese and T2D environments increase the expression and secretion of inflammatory cytokines, activation of NLRP3 inflammasome, superior migration, invasion, and phagocytosis, but have weaker immunosuppressive properties than those from lean (healthy) environments [19]. A recent study showed that intravenous administration of autologous ASCs in aged rats improved several biological aging indicators, including increasing the level of serum antioxidant enzymes [20]. However, the performance of these aging ASCs was not compared with that of young ASCs. Our in vivo comparative study of donor-matched ASCs for different recipients showed that the outcome of ASC transplantation was affected not only by the donor but also by the recipient. The transplantation outcome of different aged donor-mice indicates that only ASCs from young mice can significantly reduce the body weight and abdominal fat ratio of aged mice and effectively regulate the physiological and biochemical indexes of aged mice. However, ASC-matched with donor age had no effect on the young and ob^−^/ob^−^mice. These results suggest that ASCs derived from young mice exhibit their anti-aging effects by mainly reducing the weight and abdominal fat ratio of aged mice. Interestingly, ASCs are also effective in anti-aging treatment in D-galactose-induced aged animal models [30]. ASCs improve aging skin by increasing the expression of some angiogenesis growth factors. In line with this finding, our protein and transcriptome results show that several types of collagen genes are highly expressed in the young ASCs, but there is no difference in the expression of growth factors (including IGF-1, bFGF, VEGF) compared to the ASCs from aged donors. Previous studies show that the benefits of ASC transplantation are mainly achieved by IGF-1, bFGF, and VEGF secreted by ASCs [8, 11]. However, our results indicate that secretion of growth factors is just a consequence, not a cause. Other factors in the Y-ASCs may regulate the metabolic disorders and inflammation in the adipose tissue of aged mice. Our study showed that transplantations of Y-ASCs and O-ASCs in aged mice caused differential effects on anti-aging and weight loss. Only the ASCs from young donors have a significant impact on weight and abdominal fat loss for older recipients. Furthermore, the age-related antioxidant enzymes and hormones in the serum of aged mice treated with Y-ASCs were significantly increased but remained unchanged in aged mice treated with O-ASCs. Most importantly, the multi-omics expression profiles between Y-ASCs and O-ASCs are distinct, suggesting the underlying molecular mechanism of ASC transplantation.

In constant with other studies, our transcriptome and proteome data indicate that the differentially expressed factors between the Y-ASCs and O-ASCs are linked to cytokine secretion, fatty acid metabolism, and inflammatory response. The transcriptome of adipose tissues showed that Y-ASCs transplantation altered the abdominal fat metabolism and immune state in aged mice. To identify the potential functional modules in the multi-omics data network, we used the PPI and MCODE tools for analysis and identified Cxcl12 as a key gene. Cxcl12 is a multi-effect chemokine that participates in the regulation of tissue homeostasis, immune surveillance, inflammatory response, and cancer development. Studies have shown that Cxcl12 acts an anti-inflammatory factor when leukocytes enter the inflammatory site. This change makes the antigen-specific CD4 T cells become antigen-specific regulatory T cells (also called Tregs), which produce key anti-inflammatory cytokine IL-10 to inhibit the progress of inflammation [14]. Moreover, Cxcl12 can reduce tissue damage, inflammatory corpuscle activation and local inflammation [26], as well as improve functional recovery [27]. Our flow cytometry results indicate that Cxcl12 may play an anti-inflammatory role by inducing CD4^+^ cell polarization. Notably, Cxcl12 is identified as a hub gene in the PPI network that closely linked the upregulated genes of ASCs with the other genes after transplantation in the young mice. Cxcl12 is also highly expressed in the ASCs of young mice. However, the role of Cxcl12 in this process requires further studies.

In the past 20 years many studies have demonstrated that ASC and other stem cell transplantation offers great promise for cell therapy, but the exact mechanism is still unclear to a large extent. Since pro-inflammatory cytokines are closely involved in the regulation of inflammation, aging, obesity, and autoimmune diseases, they are likely to play important roles in organ and ASC transplantation. IL-1β, IL-6, and TNF-α are classical pro-inflammatory cytokines associated aging and obesity [6, 16, 23], the levels of IL-1β, IL-6, and TNF-α in serum were significantly decreased in the Y-ASC transplantation group when compared to the old control group. Consistent with this, The killer T cells (CD3 + CD8 +) were significantly decreased in the Y-ASC transplantation group. It is due to ASC inhibit the proliferation of lymphocytes. However, a better understanding of transplantation immunology and multimodal immunosuppression schemes can reduce the risk of failure and instability after ASC transplantation. Overall, obesity can reshape the body’s immune state and change the body’s response to immunotherapy drugs. Compared with aged ASCs, young ASCs have a stronger ability to scavenge oxidative stress and promote hormone response by promoting the high expression of antioxidant enzymes (such as SOD3), hormone receptors (such as PPARγ), and chemokines (such as Cxcl12) in young cells. At the same time, young cells possess a stronger immunosuppressive ability, which is mainly manifested in inhibiting the proliferation of CD8^+^ T cells and B cells (Table 1).

**Table 1:** List of cell typing marker proteins

Antibody	Conjugate
CD45	FITC
CD3	APC
CD4	PE
CD8	PE-Cyanine7
NK1.1	APC-eFluor 780
B220	PerCP-Cyanine5.5

## Conclusions

Our study revealed the molecular changes of adipose tissue during aging, suggesting a regulatory mechanism of adipose stem cells in the treatment of aging and senile diseases. This will not only help to enhance our understanding of the biological characteristics of adipose-derived stem cells with aging, but also lay a new theoretical and technical foundation for the clinical use of adipose-derived stem cells as a tool in the treatment of aging-related diseases.

## Supplementary Information

**Additional file 1. **The Volcano map of transcriptome and proteome of differentially expressed genes and the PPI network analysis.

**Additional file 2. **The total list of 1667 genes were differentially expressed in the Y-and O-ASCs.

**Additional file 3. **The total proteins were detected in the Y-ASCs and O-ASCs.

**Additional file 4. **The total list of 2161 genes were differentially expressed in the abdominal adipose tissue of YASC transplanted old mice compared to the control old mice.

**Additional file 5. **The Primer sequences of RT-PCR in article.

## Data Availability

The datasets generated during the current study are available from the corresponding authors upon reasonable requests. The raw sequence data reported in this paper have been deposited in the Genome Sequence Archive (Genomics, Proteomics & Bioinformatics 2021) in National Genomics Data Center (Nucleic Acids Res 2022), China National Center for Bioinformation / Beijing Institute of Genomics, Chinese Academy of Sciences (GSA: CRA008155; CRA008167) that are publicly accessible at https://ngdc.cncb.ac.cn/gsa. The data reported in this paper have been deposited in the OMIX, China National Center for Bioinformation/Beijing Institute of Genomics, Chinese Academy of Sciences (https://ngdc.cncb.ac.cn/omix: accession no.OMIX001844).

## Abbreviations

ASCs: Adipose-derived stem cells
bFGF: Basic fibroblast growth factor
GO: Gene ontology
KEGG: Kyoto encyclopedia of genes and genomes
MCODE: Molecular complex detection
ob^−^/ob^−^: Leptin knockout C57BL/6 J mice
PPI: Protein–protein Interaction
T2D: Type 2 diabetes
VEGF: Vascular endothelial growth factor
