# Supplementary material for: Anti-aging mechanism of different age donor-matched adipose-derived stem cells
Source: Stem Cell Res Ther. 2023 Aug 2;14:192. doi: 10.1186/s13287-023-03415-3 (PMC10394785; doi:10.1186/s13287-023-03415-3)
Supplement: Supplementary file 1 — Additional file 1. The Volcano map of transcriptome and proteome of differentially expressed genes and the PPI network analysis. [file 13287_2023_3415_MOESM1_ESM.docx]

**S1**

Volcano map of Y-ASCs and O-ASCs transcriptome of differentially expressed genes. Compared to the O-ASCs, 686 up-regulated and 981 down-regulated mRNAs were identified in the Y-ASCs.


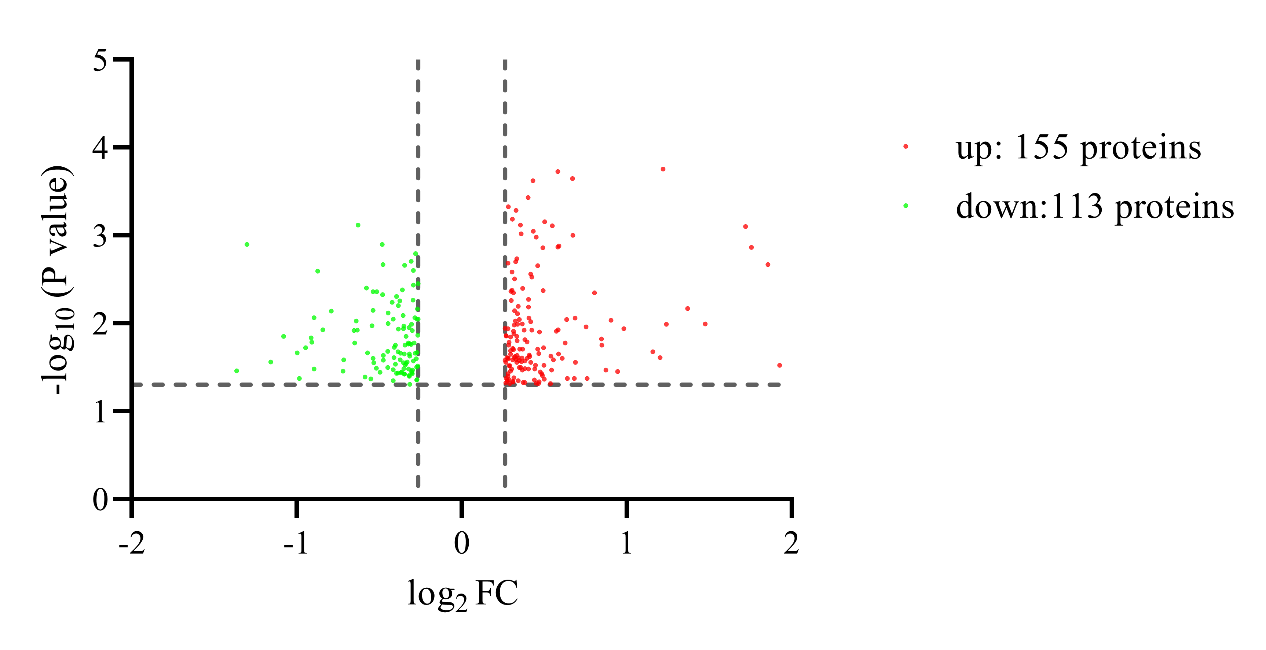


**S2**

Volcano maps of differentially expressed proteins between the Y-ASCs and O-ASCs groups. Compared to the O-ASC, 155 up-regulated and 113 down-regulated proteins were identified in the Y-ASCs.


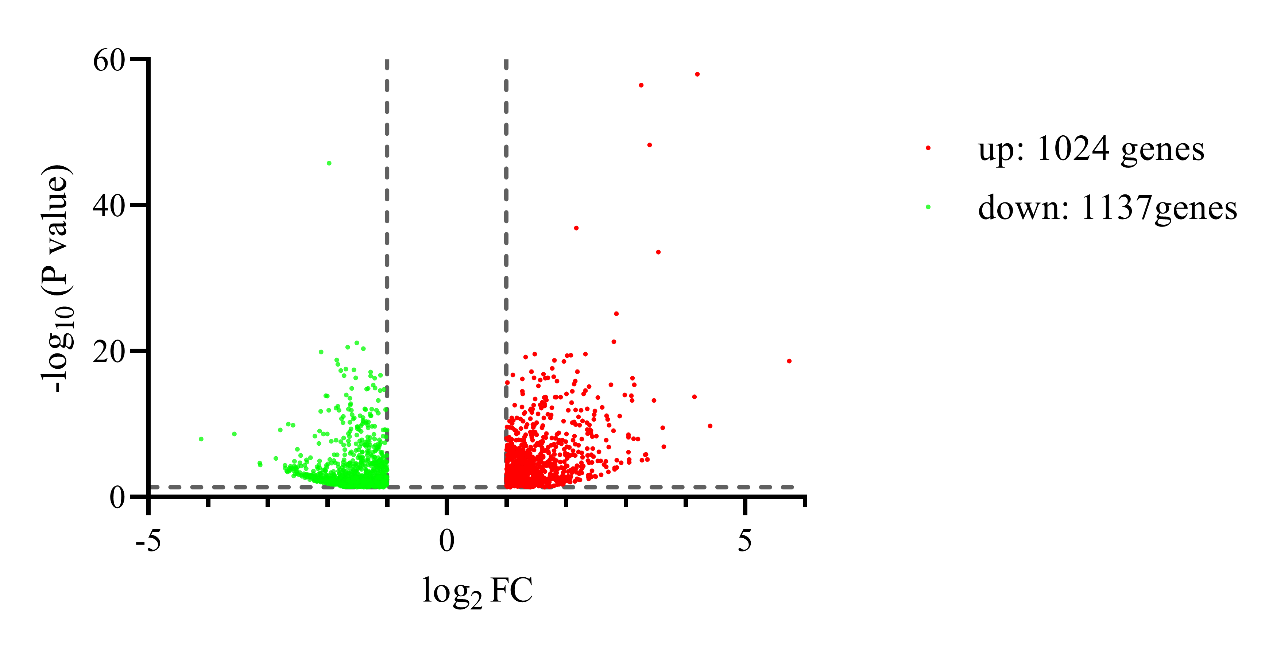


**S3**

Volcano map of differentially expressed genes in the adipose tissue of control and Y-ASCs-transplanted aged mice. Compared to the control aged mice, 1024 up-regulated and 1137 down-regulated mRNAs were identified in the abdomen adipose tissue of Y-ASC-transplanted aged mice.


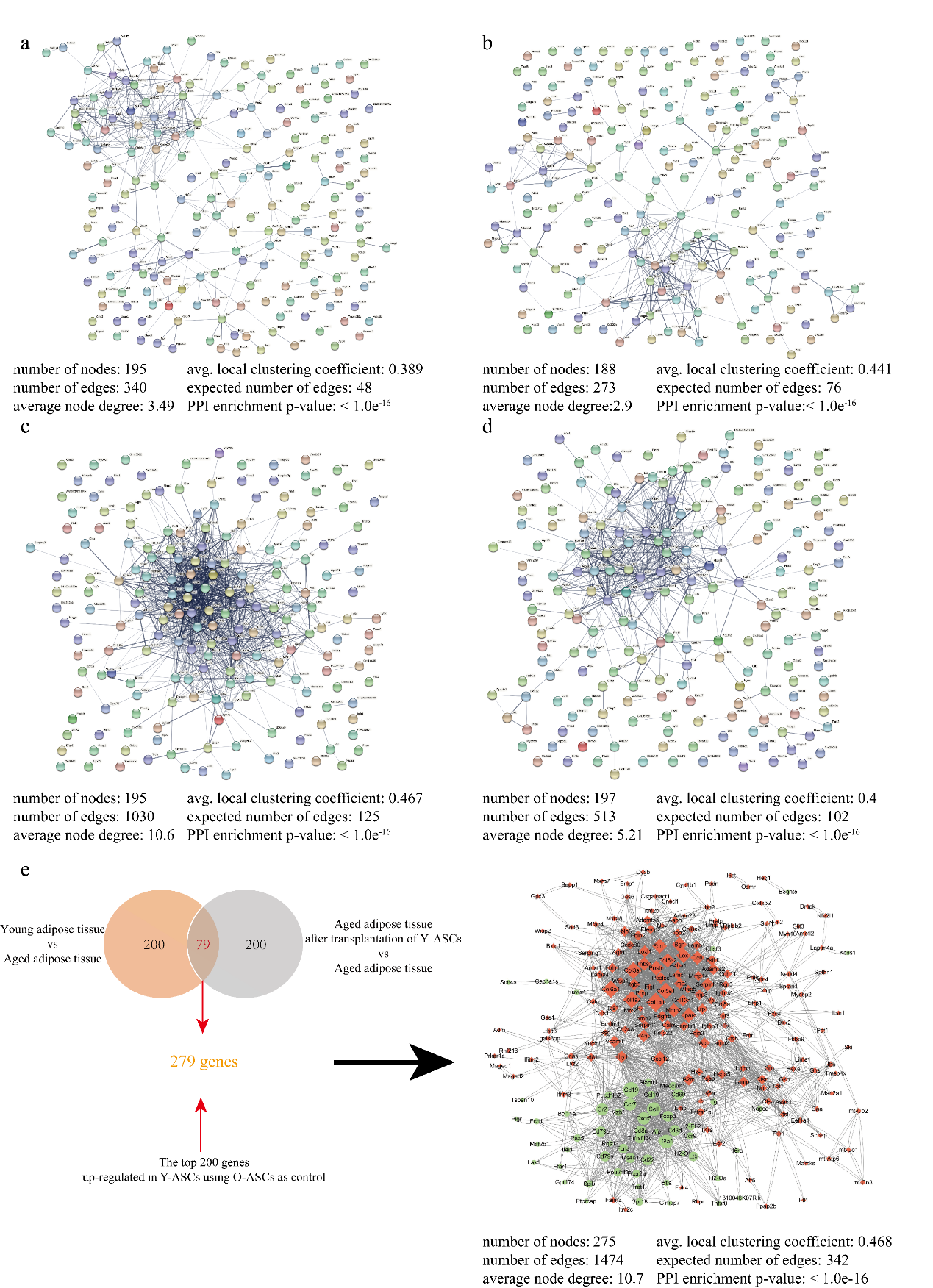


**S4 The** PPI network analysis.

**a** The PPI network analyzed with top 200 up-regulated genes in young adipose tissue versus aged adipose tissue. **b** The PPI network analyzed with top 200 up-regulated genes in the aged adipose tissue after transplantation of Y-ASCs versus aged adipose tissue. **c** The PPI network analyzed with top 200 down-regulated genes in young adipose tissue versus the aged adipose tissue. **d** The PPI network analyzed with top 200 down-regulated genes in the aged adipose tissue after transplantation of Y-ASCs versus aged adipose tissue. **e** A total of 279 proteins were screened out and constructed into the PPI network. The top 200 genes whose expressions were up-regulated in Y-ASCs using O-ASCs as control are marked as red. The 79 genes whose expressions were commonly down-regulated in the young and aged adipose tissues after transplantation of Y-ASCs using aged adipose tissues as control were marked as green.
