# Supplementary material for: Anti-aging mechanism of different age donor-matched adipose-derived stem cells
Source: Stem Cell Res Ther. 2023 Aug 2;14:192. doi: 10.1186/s13287-023-03415-3 (PMC10394785; doi:10.1186/s13287-023-03415-3)
Supplement: Supplementary file 5 — Additional file 5. The Primer sequences of RT-PCR in article. [file 13287_2023_3415_MOESM5_ESM.docx]

Table S4 Primer sequences of RT-PCR

| Name | Forward (5′→3′) | Reverse (5′→3′) |
| --- | --- | --- |
| C1qtnf5 | GAGCAACCAGAAGCTAGGACC | GCTGGGGATCTTGTTGTCGT |
| ccl2 | TGCCCTAAGGTCTTCAGCAC | AAGGCATCACAGTCCGAGTC |
| ccl7 | CGCTGCTTTCAGCATCCAAG | CTTCCCAGGGACACCGACTA |
| ccnD1 | CCCTTGACTGCCGAGAAGTT | TCATCCGCCTCTGGCATTTT |
| Col3a1 | GCGAGCGGCTGAGTTTTATG | GCAGCTCAGAGTAGCACCAT |
| F3 | AGGATGTGACCTGGGCCTAT | GTAAATGGTGGCTCCTCCCC |
| Marcks | GTGCCCAGTTCTCCAAGACC | CCCGTTCACTTTTACGTGGC |
| S100a4 | TCCTCTCTCTTGGTCTGGTCT | GTCACCCTCTTTGCCTGAGT |
| Spp1 | ATATGCGCCGCTCATCATCA | GGATGTGCTCCAGGTGTCAA |
| Wisp2 | GCGTCCGCGCTCCTG | CATCCCAAGCTACTGCCACA |
| Hmga1 | TGCGCTCCTCTAATTGGGAC | GTGCGCGGCCTCAATAAATA |
| Pcdhgc3 | AAGCGCTAACCCGCTGAAAG | GCAGAAGCAAAACTCCCACC |
| Cyp1b1 | CTGGACAAGGACGGCTTCAT | ACAGTTCCTCACCGATGCAC |
| Col8a1 | GCAAGGACTTTGGTCCTCGAT | TAGTAGGCACCGGCCTGAAT |
| Gas6 | ATGAAGATCGCGGTAGCTGG | CCAACTCCTCATGCACCCAT |
| Selenop | AAGCTAGTCCGAAGGGGTTG | CTATGTACCACTCCGGGGCT |
| Txnip | AGTTACCCGAGTCAAAGCCG | ACTGCTGAGACCCTTGCATC |
| Lpl | TTGCAGAGAGAGGACTCGGA | GTTGCACCTGTATGCCTTGC |
| Cxcl12 | CCTTCAGATTGTTGCACGGC | TCGGGGGTCTACTGGAAAGT |
